# Rectal cancer: a methodological approach to matching PET/MRI to histopathology

**DOI:** 10.1186/s40644-020-00347-6

**Published:** 2020-10-31

**Authors:** Miriam K. Rutegård, Malin Båtsman, Lennart Blomqvist, Martin Rutegård, Jan Axelsson, Ingrid Ljuslinder, Jörgen Rutegård, Richard Palmqvist, Fredrik Brännström, Patrik Brynolfsson, Katrine Riklund

**Affiliations:** 1 Department of Radiation Sciences, Diagnostic Radiology, Umeå University, Umeå, Sweden; 2 Department of Medical Biosciences, Pathology, Umeå University, Umeå, Sweden; 3 Department of Molecular Medicine and Surgery, Karolinska Institutet, Stockholm, Sweden; 4 Department of Surgical and Perioperative Sciences, Surgery, Umeå University, Umeå, Sweden; 5 Wallenberg Centre for Molecular Medicine, Umeå University, Umeå, Sweden; 6 Department of Radiation Sciences, Oncology, Umeå University, Umeå, Sweden

**Keywords:** Rectal neoplasms, EMVI, Staging, Lymph nodes, Tumour deposits

## Abstract

**Purpose:**

To enable the evaluation of locoregional disease in the on-going RECTOPET (REctal Cancer Trial on PET/MRI/CT) study; a methodology to match mesorectal imaging findings to histopathology is presented, along with initial observations.

**Methods:**

FDG-PET/MRI examinations were performed in twenty-four consecutively included patients with rectal adenocarcinoma. In nine patients, of whom five received neoadjuvant treatment, a postoperative MRI of the surgical specimen was performed. The pathological cut-out was performed according to clinical routine with the addition of photo documentation of each slice of the surgical specimen, meticulously marking the location, size, and type of pathology of each mesorectal finding. This allowed matching individual nodal structures from preoperative MRI, via the specimen MRI, to histopathology.

**Results:**

Preoperative MRI identified 197 mesorectal nodal structures, of which 92 (47%) could be anatomically matched to histopathology. Of the matched nodal structures identified in both MRI and histopathology, 25% were found to be malignant. These malignant structures consisted of lymph nodes (43%), tumour deposits (48%), and extramural venous invasion (9%). One hundred eleven nodal structures (55%) could not be matched anatomically. Of these, 97 (87%) were benign lymph nodes, and 14 (13%) were malignant nodal structures. Five were malignant lymph nodes, and nine were tumour deposits, all of which had a short axis diameter < 5 mm.

**Conclusions:**

We designed a method able to anatomically match and study the characteristics of individual mesorectal nodal structures, enabling further research on the impact of each imaging modality. Initial observations suggest that small malignant nodal structures assessed as lymph nodes in MRI often comprise other forms of mesorectal tumour spread.

**Trial registration:**

Clinical Trials Identifier**:** NCT03846882.

## Introduction

Magnetic resonance imaging (MRI) of the pelvis is fundamental to the staging and restaging of patients with adenocarcinoma of the rectum [1–4]. MRI is applied to assess markers, i.e. risk factors for increased risk of disease recurrence and metastatic disease, which are used to determine the optimal surgical and oncological treatment. These risk factors are the extent of the primary tumour (T-stage), spread to locoregional lymph nodes (N-stage), presence of extramural venous invasion (EMVI), and distance from the primary tumour to the mesorectal fascia (MRF). While most risk factors are readily evaluated with MRI, N-stage remains challenging to determine with high accuracy [2, 3, 5–8]. Moreover, lymph nodes may be difficult to differentiate from tumour deposits when normal lymph node tissue has been completely replaced by tumour. Recent studies also indicate that tumour deposits (N1c) are associated with worse survival than metastatic lymph nodes when considering both types of lesions in isolation [9–12].

In recent years, hybrid imaging with positron-emission tomography (PET) and 3 T MRI, PET/MRI, has been performed in clinical practice as an alternative to PET/computed tomography (CT) when better soft-tissue contrast is of importance, e.g. in the head-and-neck region, brain, pelvis, breast, and in paediatric oncology [13, 14]. Few studies have evaluated the role of ^18^F-fluoro-2-deoxy-D-glucose (FDG) PET/MRI in colorectal cancer and even fewer in patients with primary rectal cancer [15–20]. None of these studies have matched individual nodal structures anatomically with a histopathological analysis, which is of importance for a valid comparison of imaging and histopathology.

The aim of the present study is to present a method for anatomical matching of individual mesorectal structures between preoperative MRI and histopathology as part of the on-going prospective RECTOPET (REctal Cancer Trial on PET/MRI/CT) study. This development enables later assessment of the metabolic and morphological characteristics of mesorectal nodal structures on a finding-by-finding basis using FDG-PET/MRI.

## Materials and methods

### Patient population

**Fig. 1:**
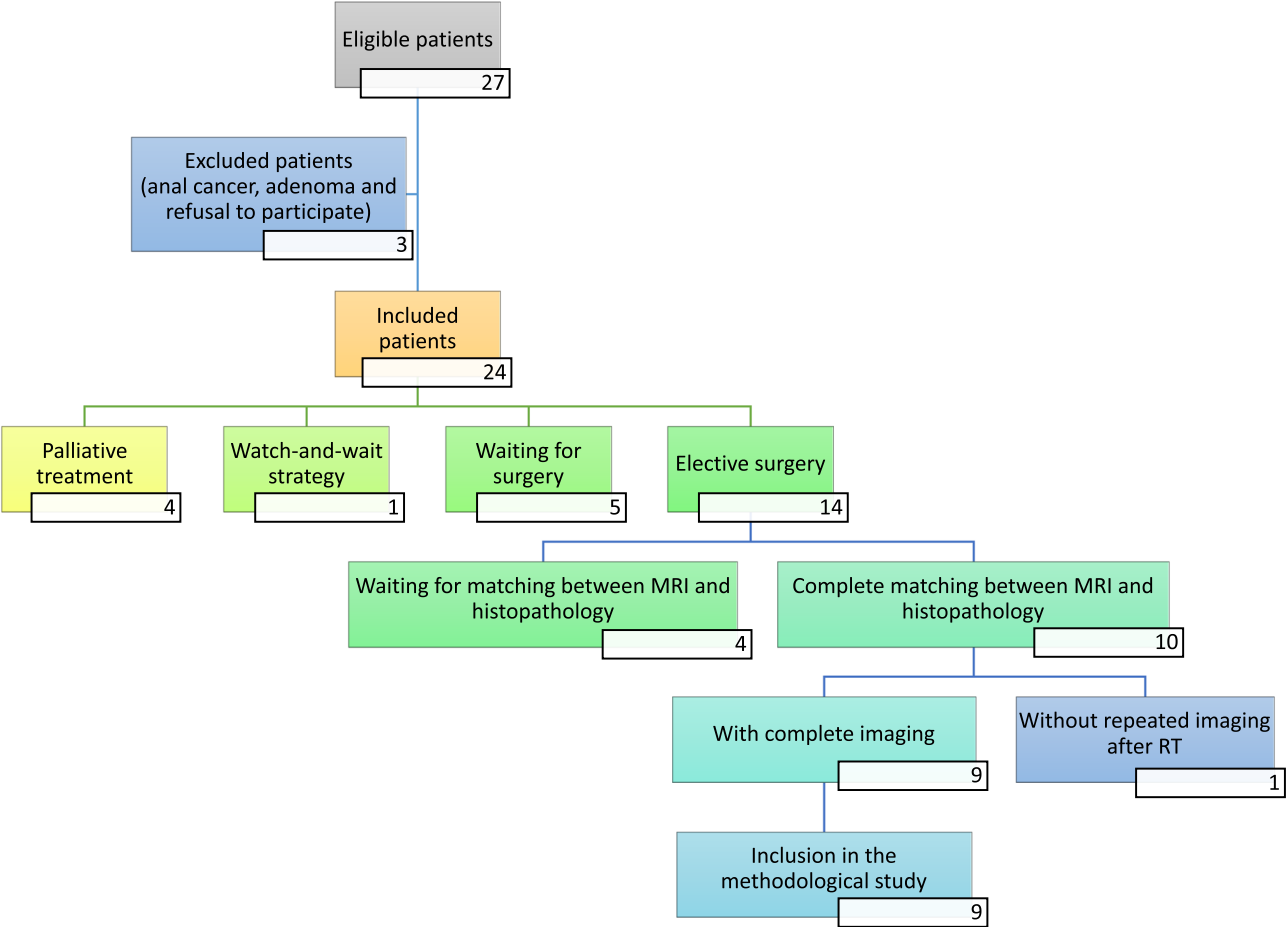
Flowchart for the RECTOPET study and for the patients that were included in the methodological study. The included patients all had a complete set of staging (and restaging if administered neoadjuvant treatment), as well as postoperative specimen examinations, and a complete matching of nodal structures between MRI and histopathology. RT = Radiotherapy (Small boxes show the number of patients)

This methodological study is based on the patients included in the prospective observational cohort study, RECTOPET (REctal Cancer Trial on PET/MRI/CT [NCT03846882]). The inclusion criteria for the ongoing RECTOPET study, as well as a flow chart presenting the path of the included patients through the various examinations, have been published elsewhere [21]. In summary, patients included in the RECTOPET study had a biopsy-proven rectal cancer and underwent a staging FDG-PET/CT and FDG-PET/MRI. If neoadjuvant treatment was given with the intention of delayed surgery [2], a restaging FDG-PET/CT and FDG-PET/MRI was done six to eight weeks after the completed neoadjuvant treatment. If the patient subsequently underwent tumour resection, an MRI of the surgical specimen was performed postoperatively, and finally, histopathological analysis with finding-to-finding anatomical matching was done, as described in the sections *Identification, matching, and characterisation of nodal structures between patient MRI and specimen* and *Finding-by-finding MRI-histopathology matching* below. The present methodological study includes only patients who had a complete set of staging (and restaging if administered neoadjuvant treatment) as well as postoperative specimen examinations and an anatomical matching of nodal structures between MRI and histopathology. Therefore, patients with advanced disease selected for non-operative palliative treatment and patients without restaging imaging after radiotherapy were excluded (Fig. 1).

### Patient characteristics

Nine patients had been included as of December 2017 (Fig. 1 and Table 1). The neoadjuvant treatment group consisted of five patients: three treated with radiotherapy ([RT]; 5 × 5 Gy) and delayed surgery (7–18 weeks after completion of RT), and two treated with chemoradiotherapy ([CRT]; 1.8 × 28 Gy to 50.4 Gy) and delayed surgery (8–10 weeks after completion of RT). The median time from completion of neoadjuvant treatment to restaging PET/MRI was 7 weeks (range: 6–8 weeks). The remaining patients (*n* = 4) all underwent primary surgery without neoadjuvant treatment.

**Table 1:** Clinical data for the 9 patients included in the methodological part of the RECTOPET study

Variables	N	%
**Sex**		
Male	6	67
Female	3	33
**Tumour height**		
≤ 5 cm	3	33
6–10 cm	4	44
11–15 cm	2	22
**cT-stage**		
1	0	0
2	3	33
3	6	67
4	0	0
**cN-stage**		
0	6	67
1	1	11
2	2	22
**cM-stage**		
0	8	89
1	0	0
Undetermined	1	11
**Management**		
Primary surgery	4	44
Preoperative radiotherapy	3	33
Preoperative chemoradiotherapy	2	22
**pT-stage**		
0	0	0
1	1	11
2	2	22
3	6	67
4	0	0
**pN-stage**		
0	4	44
1	4	44
2	1	11

### Staging and restaging MRI

Preoperative MRI of the pelvis was performed with a 3 T PET/MRI (SIGNA PET/MRI, GE Healthcare, Milwaukee, WI). A phased-array surface coil was used in a standardised protocol consistent with the European Society of Gastrointestinal Radiology (ESGAR) consensus guidelines [2]. An intravenous antiperistaltic agent, 40 mg of butylscopolamine bromide (Buscopan, Boehringer Ingelheim), was used, and if contraindicated, 1 mg (1 IE) of glucagon (Glucagon, Novo Nordisk) was used instead as a subcutaneous injection. The standard pulse sequences consisted of multiplanar 2D T2-weighted fast spin-echo (transaxial, sagittal, and coronal) with 3–4-mm slice thickness, a high-resolution oblique sequence perpendicular to the rectal tumour with a slice thickness of 3 mm, and a transaxial diffusion-weighted sequence (b = 200 and b = 800 s/mm2, including an ADC map). In addition, the protocol included a transaxial 3D T1-weighted spoiled gradient-echo sequence with a 1-mm slice thickness covering the pelvic region to facilitate the identification of nodal structures. MRI parameters are shown in Table 2.

**Table 2:** Protocol parameters for the preoperative MRI

Sequence	TR [ms]	TE [ms]	ETL	FOV [mm]	Slice thick. /gap [mm]	NEX	Matrix	Pixel [mm]	Acq. time	BW [Hz/px]
**Sag T2 FRFSE**	3900	102	20	200 × 200	3.0/0.0	3	320 × 320	0.63 × 0.63	04:33	223
**Ax T2 FRFSE**	5719	100	16	270 × 270	4.0/0.4	1	384 × 256	0.70 × 1.05	03:38	325
**Cor T2 FRFSE**	4000	102	24	220 × 220	3.0/0.0	3	320 × 320	0.69 × 0.69	04:16	260
**T2 perp (ax)**	4000	100	16	210 × 210	3.0/0.0	2	384 × 256	0.55 × 0.82	04:32	260
**Ax DWI Focus**	3500	69.4	–	240 × 120	4.0/0.0	1	160 × 80	1.5 × 1.5	04:47	3125
**Ax T1 FSPGR**	4.7	1.9	–	256 × 256	1	1	256 × 256	1.0 × 1.0	04:53	488

### MRI of the surgical specimen

The surgical specimen was placed in a plastic box and collected from the pathology unit after fixation in formaldehyde, as described in the section *Histopathological diagnostics and finding-by-finding description* below. The plastic box with the specimen was scanned using a flex-array coil (upper anterior array). The protocol used is presented in Table 3 and consisted of a 3D T1-weighted sequence covering the entire specimen with a 0.5-mm slice thickness and a 2D T2-weighted sequence in the sagittal plane with a slice thickness of 2 mm.

**Table 3:** Protocol parameters for MRI of the surgical specimen

Sequence	TR [ms]	TE [ms]	ETL	FOV [mm]	Slice thick. /gap [mm]	NEX	Matrix	Pixel [mm]	Acq. Time	BW [Hz/px]
**Cor T1 FSPGR**	10.3	2.7	–	256 × 154	0.5	2	512 × 307	0.50 × 0.50	15:51	488
**Sag T2 FRFSE**	9906	68	16	200 × 120	2.0/0.0	6	352 × 211	0.57 × 0.57	14:02	237

### Histopathological diagnostics and finding-by-finding description

The pathology department received the surgical specimens fresh. The specimens were examined macroscopically on arrival to evaluate the resection plane [22]. The whole surgical specimen was inked for later orientation, and the anterior aspect of the specimen was opened, except for the area above and adjacent to the tumour. A foam was passed through the residual lumen to improve fixation of the tumour region. The specimen was then fixated in formaldehyde for 2 days.

The first cut-out was performed by cutting the whole specimen in approximately 5-mm thick slices perpendicular to the bowel lumen. All slices were photographed. At least one whole slice with a macroscopic tumour and four selected tissue blocks containing tumour growth were embedded for microscopic evaluation. After the cut-out, the slices were packaged in gauze in numerical order from the aboral to the oral end. The packages were, subsequently, placed in a GEWF solution (Glacial acetic acid, Ethanol, distilled Water, and Formaldehyde) for at least 48 h to optimize the lymph node yield [23].

**Fig. 2:**
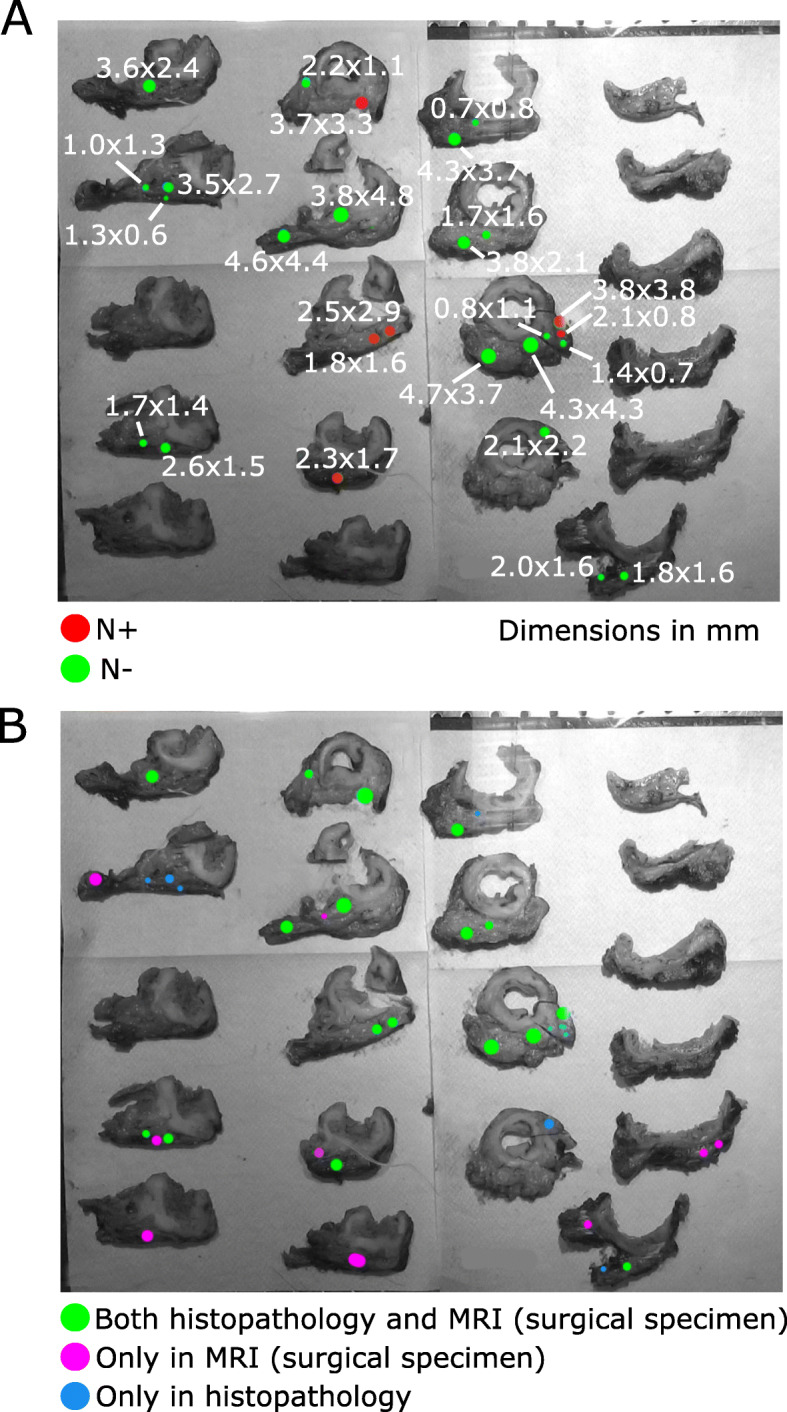
**a** Surgical specimen sliced and photographed providing a finding-by-finding description where individual nodal structures are illustrated according to size, location, and type of pathology. N- in green denotes a benign lymph node, while N+ in red indicates a malignant lymph node. No other nodal structures, such as tumour deposits, were found in these 20 slices. **b**. The same finding-by-finding description as in (**a**) after joint comparison and consensus between the study radiologist and pathologist. Green colour indicates anatomically matched lymph nodes between histopathology and MRI of the surgical specimen; blue signifies lymph nodes found at histopathology but not in MRI of the surgical specimen; pink denotes lymph nodes found in MRI of the surgical specimen but not at histopathology

Each slice was subdivided and thoroughly examined for discrete free-laying structures within the perirectal adipose tissue, presumed to represent lymph nodes or tumour manifestations. The pathologist documented the slice number, the position, and the relative size of each nodal structure for each finding. The nodal structures were also inked to aid identification at the subsequent microscopic examination, if needed. All glass slides were stained with haematoxylin and eosin staining. Elastin staining was performed to facilitate identification of EMVI [22]. Microscopic evaluation was performed according to the recommendations from the World Health Organization and Tumour-Node-Metastasis (TNM) classification system, 7th edition [24, 25]. Collections of tumour cells found in the perirectal tissue where no residual vessel wall, nerve structures, or recognisable lymphoid tissue could be found was categorised as tumour deposits, in accordance with the TNM system. Lymph nodes and lymphoid aggregates were differentiated according to Swedish national guidelines [26], which state that a lymph node should have a peripheral sinus. A resident in pathology (M.B.) assessed and recorded the type, location, size, and presence of tumour cells of each nodal structure, hence, creating a finding-by-finding description as shown in Fig. 2a. The sizes were measured in two dimensions on the glass slides, and the shorter of these measurements are herein called the short axis. Of note, lymph nodes from the lateral compartment were not included since they were not part of the surgical specimen, as lateral lymph node dissection was not performed at Umeå University Hospital at the time of inclusion.

### Identification, matching, and characterisation of nodal structures between patient MRI and specimen MRI

**Fig. 3:**
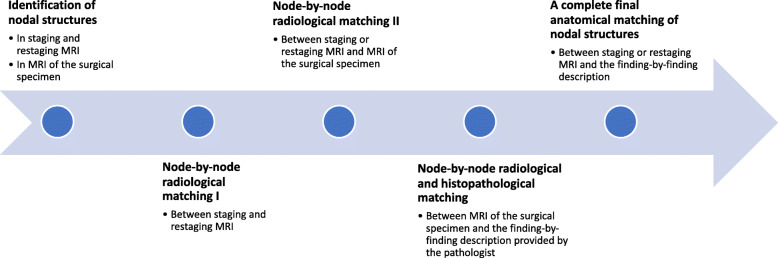
Flowchart of the process of matching individual nodal structures anatomically

All nodal structures (lymph nodes, tumour deposits, or EMVI) in the mesorectum and directly above the mesorectum, along the mesorectosigmoidal chain, are herein called mesorectal nodal structures. The anatomical matching of mesorectal nodal structures between the staging and restaging MRI, the MRI of the surgical specimen, and the finding-by-finding description was done by the study radiologist (M.K.R.), a resident in radiology. The work process is visualised in a flowchart in Fig. 3.

First, mesorectal nodal structures recognised as lymph nodes radiologically (isolated mesorectal structure with a round or oval shape, not continuous with a vessel or the primary tumour) were located in the staging MRI; suspicious EMVI was also noted but not included in the count of nodal structure and was not included in the matching process for the nodal structures. Second, if relevant, the same nodal structures were located in the restaging MRI. Third, all of the mesorectal nodal structures were located in the MRI of the surgical specimen and, when possible, matched anatomically to the previous staging or restaging MRI. In this step, additional nodal structures could, in some cases, be found in the staging or restaging MRI with the aid of the MRI of the surgical specimen. Each mesorectal nodal structure was localised using the transaxial T1-weighted sequence combined with the diffusion-weighted sequence. Each nodal structure was then localised in T2-weighted sequences in all three planes to avoid confusion with vessels. Size, including both short and long axis diameter (mm), was recorded as well as morphological MRI features; these comprised shape (indistinct/irregular margin, and round) and signal intensity (homogenous/heterogenous), all to be presented in later reports.

### Finding-by-finding MRI-histopathology matching

**Fig. 4:**
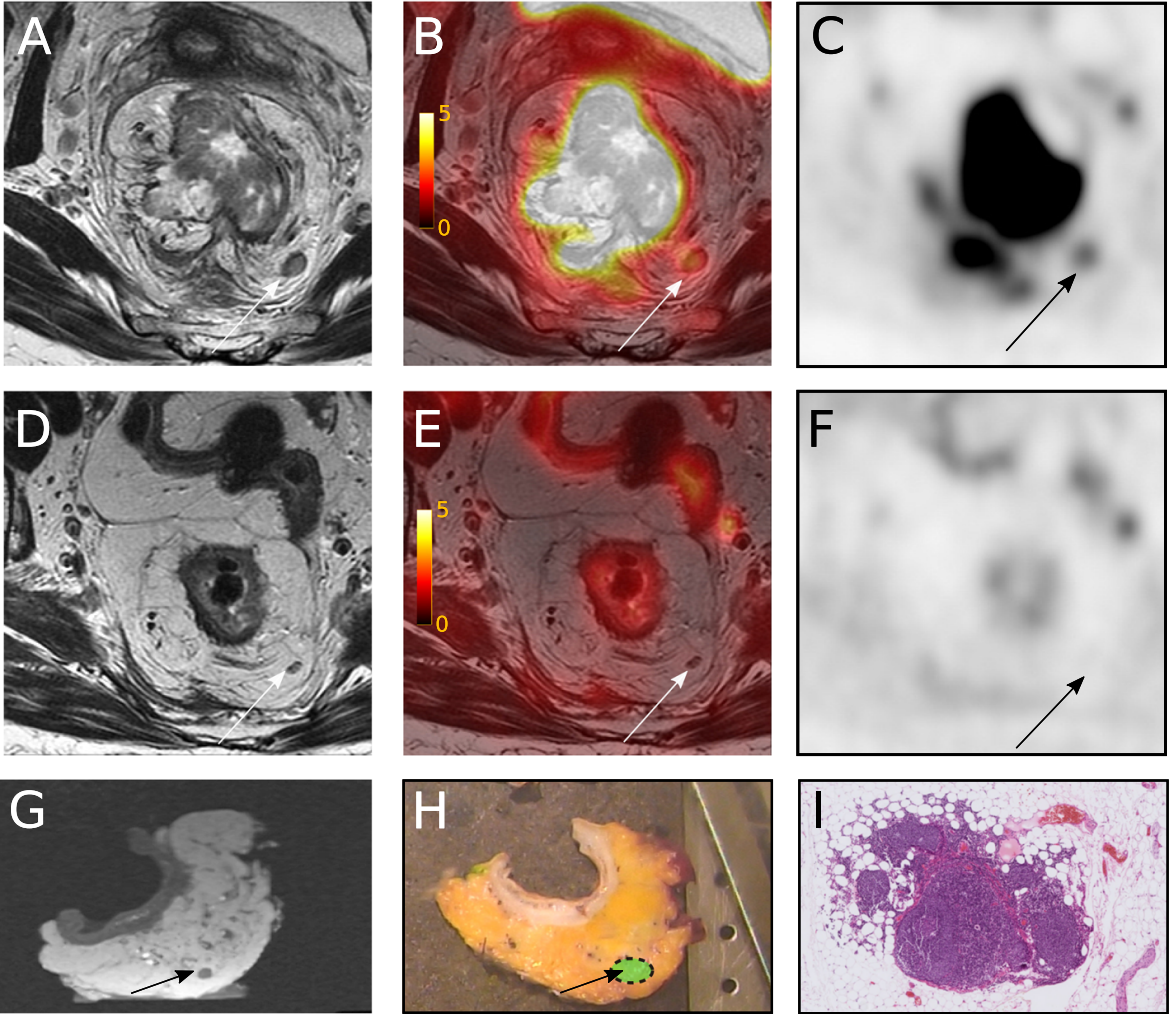
**a-i**. Patient in the neoadjuvant treatment group with mucinous rectal cancer. **a-c**: Staging FDG-PET/MRI before chemoradiotherapy. Anatomically matched nodal structure measuring 6.3 mm in short axis seen in a) transaxial T2-weighted sequence perpendicular to the tumour, b) FDG-PET/MRI with a T2-weighted MR sequence showing increased metabolic activity (above background) in the same nodal structure, and c) Static 3D MAC PET image. **d-f**: Restaging FDG-PET/MRI after chemoradiotherapy in the same patient. The selected and anatomically matched nodal structure now measures 2.9 mm in the short axis, seen in d) transaxial T2-weighted sequence perpendicular to the tumour and e) FDG-PET/MRI with a T2-weighted MR sequence, with no residual metabolic activity, and f) Static 3D MAC PET image. **g-i**: The same nodal structure in g) transaxial T1-weighted sequence MRI of the surgical specimen, h) in the finding-by-finding description using the photographed slices arrayed numerically, and i) at microscopy, using hematoxylin & eosin stain at 5x magnification where no residual malignant growth was seen. *Including the two nodal structures that proved to be EMVI at histopathology analysis. EMVI was not measured to size and is, therefore, not accounted for in the histopathological nodal structures

Using the pathologist’s descriptions, the study radiologist performed an independent matching of mesorectal nodal structures between the finding-by-finding description and the MRI of the surgical specimen. Together, the pathologist and radiologist then verified the findings, and the nodal structures for which consensus was found were further matched with staging and restaging MRIs. Nodal structures assessed as lymph nodes in MRI could, in this stage, prove to be another type of finding when individually matched with histopathology; such discrepancies were noted. An example of a finding-by-finding description before and after joint comparison and consensus between the study radiologist and pathologist is shown in Fig. 2a and b. An example of an anatomically matched nodal structure from identification in staging and restaging FDG-PET/MRI to assessment in microscopy is shown in Fig. 4a-i.

## Results

**Fig. 5:**
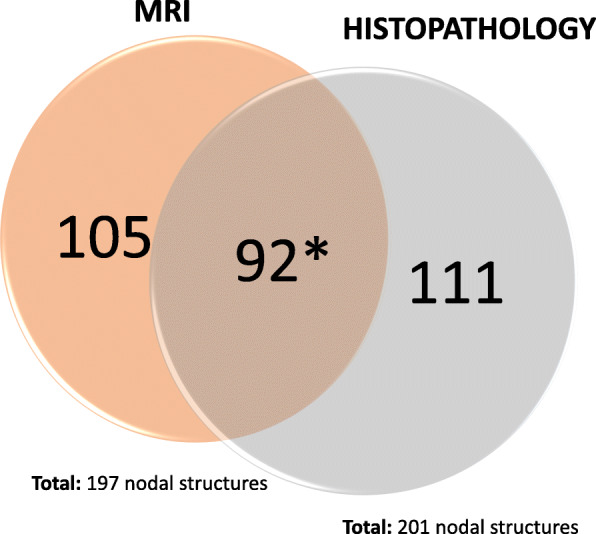
All mesorectal nodal structures found in staging MRI and histopathology and the number of nodal structures that could be anatomically matched between MRI and histopathology

**Fig. 6:**
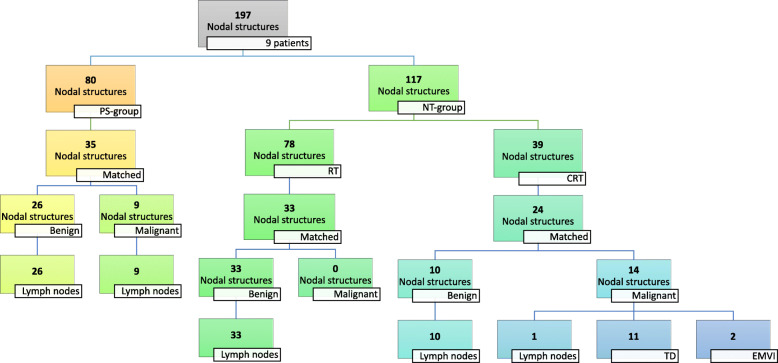
Nodal structures found in staging MRI of all the patients included in the methodological study divided into a primary surgery (PS) group and a neoadjuvant treatment (NT) group, as well as the distribution of nodal structures anatomically matched between MRI and histopathological analysis. Nodal structures denote lymph nodes, tumour deposits (TD), and extramural venous invasion (EMVI) assessed as lymph nodes in staging MRI. RT = Radiotherapy, CRT = Chemoradiotherapy

**Fig. 7:**
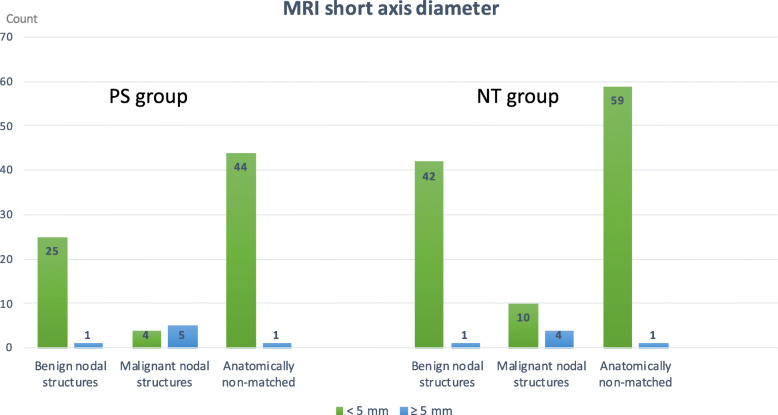
Short axis diameter of the anatomically matched, i.e. the benign and malignant nodal structures in each group, and the anatomically non-matched nodal structures between MRI and histopathology according to staging and restaging MRI for all included patients divided into a primary surgery (PS) group and a neoadjuvant treatment (NT) group. The nodal structures are stratified in histopathologically verified malignant and benign nodal structures, where all of the benign nodal structures were found to be benign lymph nodes at histopathological analysis. The non-matched nodal structures in this figure are the non-matched nodal structures found in staging and restaging MRI

**Fig. 8:**
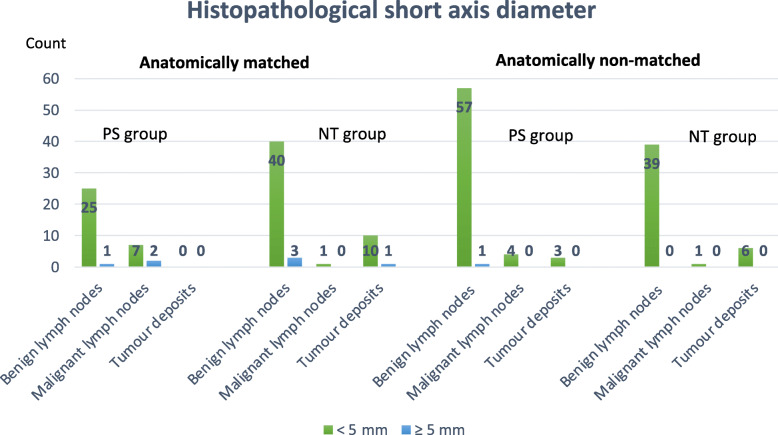
Short axis diameter of all of the anatomically matched and non-matched nodal structures between MRI and histopathology in all nine patients, according to histopathology, divided into a primary surgery (PS) group and a neoadjuvant treatment (NT) group. The size of extramural venous invasion [EMVI] was not measured in the histopathological analysis and is, therefore, not accounted for in this figure. Benign nodal structures are, in this context, called benign lymph nodes since this was histopathologically verified. The anatomically non-matched nodal structures in this figure are the histopathologically non-matched lymph nodes and tumour deposits

One hundred ninety-seven nodal structures were found in staging and restaging MRIs, and 201 nodal structures were found at histopathology. As shown in Fig. 5, 92 (47%) of the nodal structures were anatomically matched between MRI and histopathology, but only 90 were measured at the histopathological analysis since two of the structures that on glass slides seemed like nodal structures proved to be EMVI. The MRI median short axis diameter for the matched nodal structures was 2.9 mm (range: 1.0–7.6 mm). Histopathologically, the median short axis size for the matched nodal structures was 2.2 mm (range: 0.2–6.3 mm). The number of anatomically matched mesorectal nodal structures between staging or restaging MRI and histopathology in all the included patients, divided into a primary surgery (PS) group and a neoadjuvant treatment (NT) group, is presented in Table 4 and Fig. 6. Figure 6 also shows the distribution of the histopathologically benign and malignant nodal structures, as well as the histologically verified nature of the nodal structures identified in the preoperative MRI divided into the PS and the NT groups. The sizes and distribution of the anatomically matched and non-matched nodal structures between staging or restaging MRI and histopathology are shown in Figs. 7 and 8, according to PS or NT allocation. Figure 7 presents these results from a radiological perspective, while Fig. 8 displays the data from a histopathological perspective.

**Table 4:** Anatomically matched nodal structures in the primary surgery group and the neoadjuvant treatment group

	Primary surgery	Neoadjuvant treatment and restaging MRI	Total
**Number of patients**	4	5	9
**Nodal structures in staging MRI**	80	117	197
**Nodal structures in restaging MRI**	N/A	117	117
**Nodal structures in MRI of surgical specimen**	176	166	342
Nodal structures at histopathology*	100	101	201
**Lymph nodes at histopathology**	97	85	182
**Nodal structures matched between staging or restaging MRI and MRI of surgical specimen**	64	92	156
**Nodal structures matched between MRI of surgical specimen and histopathology**	77	78	155
**Nodal structures matched between staging or restaging MRI and histopathology**	35	57	92
Malignant nodal structures at histopathology*	16	19	35
**Malignant nodal structures matched between staging or restaging MRI and histopathology**	9	14	23
**Malignant lymph nodes at histopathology**	13	2	15
**Malignant lymph nodes matched between staging or restaging MRI and histopathology**	9	1	10

For all nine included patients, both in the PS group and the NT group, 23/92 anatomically matched nodal structures were histopathologically verified malignant nodal structures (66% of all malignant nodal structures found at histopathology). All of the malignant nodal structures were radiologically assessed as lymph nodes; histopathologically 11/23 (48%) were proven to be tumour deposits, 10/23 (43%) were malignant lymph nodes, and 2/23 (9%) were EMVI. The malignant structures had an MRI median short axis diameter of 4.1 mm (range: 2.2–6.9 mm) and a histopathological median short axis diameter of 2.5 mm (range: 0.2–6.3 mm). 14/23 (61%) structures had an MRI short axis size < 5 mm, while 9/23 (39%) had an MRI short axis size ≥ 5 mm. Histopathologically, 13/21 (62%, EMVI not included) of the malignant nodal structures had a short axis size < 5 mm. This is shown in Figs. 7 and 8 divided into the PS and the NT groups. All of the patients with a locoregional spread had the presence of malignant nodal structures with a short axis size < 5 mm; these were two patients in the PS group and two patients in the NT group.

### Primary surgery group

As shown in Table 4 and Fig. 6, 80 mesorectal nodal structures were identified in staging MRI, and 100 mesorectal nodal structures were identified at histopathology in the primary surgery group. The number of anatomically matched nodal structures between staging/restaging MRI and histopathology were 35 (44% of the nodal structures found in MRI and 35% of the nodal structures found at histopathology). The MRI median short axis diameter for the 35 anatomically matched nodal structures was 3.2 mm (range: 1.2–6.4 mm). Histopathologically, the median short axis size was 2.4 mm (range: 0.4–6.3 mm). The total number of matched malignant nodal structures was 9/16 (56% of the total number of malignant nodal structures found at histopathology in the PS group), all radiologically assessed as lymph nodes. The MRI short axis of the lymph nodes was < 5 mm in 4 (44%) and ≥ 5 mm in 5 (56%). All were histopathologically verified malignant lymph nodes, and histopathologically, seven (78%) of the malignant lymph nodes had a short axis size < 5 mm.

### Neoadjuvant treatment group

As shown in Table 4 and Figs. 6, 117 mesorectal nodal structures were identified in staging MRI, and 101 mesorectal nodal structures were identified at histopathology in the neoadjuvant treatment group. The number of anatomically matched nodal structures between staging/restaging MRI and histopathology were 57 (49% of the nodal structures found in MRI and 56% of the nodal structures found at histopathology). The median short axis diameter in the staging MRI was 3.3 mm (range: 1.1–8.4 mm), and the median short axis diameter in the restaging MRI was 2.2 mm (range: 1.0–6.9 mm). The median short axis size was 2.2 mm (range: 0.2–6.1 mm) at histopathology. The number of matched malignant nodal structures was 14/19 (74% of the malignant nodal structures found at histopathology in the NT group), of which one (7%) was a malignant lymph node, 11 (79%) were tumour deposits, and two (14%) were EMVI. Of these malignant nodal structures, 10 (71%) had an MRI short axis size < 5 mm, and 4 (29%) had an MRI short axis size ≥ 5 mm. The malignant lymph node and ten of the tumour deposits (92% of the matched malignant nodal structures found at histopathology) had a short axis size < 5 mm; the two nodal structures that proved to be EMVI were not accounted for, as explained initially in the *Results* section.

### Nodal structures not anatomically matched between staging MRI and histopathology

One-hundred-and-five of the nodal structures (53%) found in the staging MRI in both the PS and the NT groups could not be matched anatomically to histopathology, as seen Fig. 7. Of these, 103 (98%) had a short axis diameter < 5 mm, and the median short axis diameter was 1.6 mm (range: 0.8–6.2 mm). The MRI short axis size of the anatomically non-matched nodal structures is presented in Fig. 7, divided into the PS and the NT groups. At histopathology, 111 nodal structures (55%) could not be matched anatomically to staging or restaging MRI with a median short axis diameter of 1.3 mm (range: 0.4–5.3 mm) as presented in Fig. 8, according to PS or NT allocation. Of these, 97 (87%) were benign lymph nodes, 14 (13%) were malignant nodal structures, five were malignant lymph nodes, and nine were tumour deposits, all of which had a short axis diameter < 5 mm with a median short axis diameter of 1.7 mm (range: 0.4–4.3 mm). Seven were found in the PS group and seven in the NT group.

### Anatomical matching of EMVI

Three of the nine patients were found to have EMVI at histopathology in a separate matching of findings with suspicion of EMVI. However, only two patients had anatomically matched EMVI findings between staging or restaging MRI and histopathology in this study: one patient in the primary surgery group, and one patient in the neoadjuvant group, treated with CRT (the latter had several EMVI findings at histopathology, and the individual anatomical matching was, therefore, less certain).

## Discussion

MRI is of major importance when deciding optimal treatment strategies for rectal cancer patients. In this methodological study, we were able to match mesorectal nodal structures anatomically on preoperative MRI with dedicated histopathological analysis, thus devising a method to comprehensively study the MRI characteristics of benign and malignant nodal structures, including tumour deposits and EMVI on a finding-by-finding basis.

Attempts to develop improved accuracy are desirable as the assessment of N-stage remains challenging in rectal cancer [2, 3, 5–8]. In a recent study, Baily et al. show that PET/MRI identified 94 hypermetabolic nodal structures presumed to be lymph nodes in 29 patients with rectal cancer, where approximately 61% of the metabolically abnormal lymph nodes had a size of 5 mm or less [20]. While the study lacked histopathological matching, the findings suggest that the assessment of mesorectal nodal structures could be facilitated with the aid of the metabolic information from FDG-PET/MRI. One of the objectives of the on-going prospective RECTOPET study is to investigate the role of FDG-PET/MRI in the preoperative locoregional staging and restaging in rectal cancer; a feasible method for anatomical matching of mesorectal structures between preoperative MRI and histopathology is a cornerstone in enabling evaluation of hybrid imaging in rectal cancer. About half of the nodal structures radiologically assessed as lymph nodes in the present study were anatomically matched to histopathology, hence providing data to evaluate the efficacy of MRI and PET/MRI in identifying perirectal malignant spread in future reports.

The results from this study also indicate that there is a discrepancy regarding the characterisation of small (< 5 mm) mesorectal nodal structures in preoperative MRI compared to histopathology, indicating that MRI cannot reliably discern between tumour deposits and lymph node metastasis. Furthermore, of the malignant lymph nodes and tumour deposits in the neoadjuvant treatment group, 71% had an MRI short axis size of < 5 mm, and none had a short axis size above 9 mm. This is an important finding as it may indicate a possible limitation in the current guidelines for MRI assessment at restaging established by the 2016 European Society of Gastrointestinal and Abdominal Radiology (ESGAR) consensus meeting, which state that ‘all nodes with a short axis diameter < 5 mm [after neoadjuvant treatment] should be considered benign’ [2].

In the literature, tumour deposits are known to be misinterpreted as metastatic lymph nodes based on imaging [27, 28]. Tumour deposits have been included in the TNM classification system since the 7th edition by the addition of the N1c-stage, which is defined as the presence of tumour cells in the mesorectal fat without any concurrent lymph node metastases [25, 29]. However, a recent meta-analysis has shown that tumour deposits are associated with a worse prognosis than isolated lymph node metastases and EMVI, considering disease-free survival and that the combination of tumour deposits and lymph node metastasis are a strong predictor for metastatic disease [9]. According to the authors of the meta-analysis, this is not taken into consideration in today’s TNM criteria [9]. The number of tumour deposits in the present study exceeded the number of malignant lymph nodes, highlighting the need to properly identify and elucidate the prognostic role of these lesions.

A strength of the present methodological study is the relatively high success rate of finding-by-finding matching, particularly among the larger nodal structures where the median MRI size of the matched structures was 2.9 mm, compared with 1.6 mm in the non-matched nodal structures in MRI. This indicates that the matching procedure is able to include most of the malignant nodes, as previous studies have shown that although metastasis can occur in smaller lymph nodes, they are more likely to occur in nodes of larger sizes [30, 31].

There are a number of weaknesses to this methodological study. First, there was a limited number of patients, even though the total number of anatomically matched nodal structures between staging or restaging MRI and histopathology was comparatively large. Second, only 66% (23/35) of the malignant nodal structures found at histopathology (EMVI not accounted for) could be anatomically matched between staging or restaging MRI and histopathology. These difficulties might be explained by the small size of the missed lymph nodes and tumour deposits, where all but one measured less than 4 mm in diameter. Most of the non-matched malignant nodes were also found in the group of patients who had received neoadjuvant treatment, which is known to cause shrinkage of lymph nodes [32, 33]. Third, there was a discrepancy between the total number of nodal structures found in the MRI of the surgical specimens compared to histopathology (342 versus 201 nodal structures). This could be explained by the higher resolution of the specimen MRI (0.5 mm axial slices) compared to the in vivo MRI (1 mm axial slices, including patient movement and a larger imaging volume). Most of the non-matched structures measured less than 1 mm in size and were thus, as stated in the section *Nodal structures not anatomically matched between staging MRI and histopathology*, difficult to accurately classify using MRI. The findings are consistent with a recent study that showed that MRI visualises more lymph-node-like structures than even MRI-guided histopathology [34]. The MRI of the specimen in both our and the previously mentioned study was, however, acquired from formaldehyde fixated tissue, and to the best of our knowledge, there have been no reports on how this might alter MRI findings.

Of note, some of the discrepancy between the MRI and histopathological short axis size found in this study can be explained by the fact that the microscopy assessment was done on a thin (4 μm) slice of the nodal structure, and it is difficult to capture the real centre of the node. In some cases, the MRI nodal structure was also found to be two or three nodal structures at histopathology, and in those instances, the nodal count was based on the histopathological assessment.

## Conclusion

In conclusion, this study showed that an anatomical finding-by-finding matching of mesorectal structures between preoperative MRI and histopathology is possible. This establishes a framework within which the impact of PET/MRI on the preoperative imaging of primary rectal cancer can be evaluated and to determine the actual nature of nodal structures radiologically assessed as lymph nodes.

## Data Availability

The datasets used and/or analyzed during the current study are available from the corresponding author on reasonable request.

## Abbreviations

CT: Computed tomography
CRT: Chemoradiotherapy
EMVI: Extramural venous invasion
ESGAR: European Society of Gastrointestinal and Abdominal Radiology
FDG: ^18^F-fluoro-2-deoxy-D-glucose
GEWF: Glacial acetic acid, Ethanol, distilled Water and Formaldehyde
MRF: Mesorectal fascia
MRI: Magnetic resonance imaging
PET: Positron-emission tomography
RECTOPET: REctal Cancer Trial on PET/MRI/CT
RT: Radiotherapy
TNM: Tumour-Node-Metastasis (TNM) classification system

## References

[1] Jhaveri KS, Hosseini-Nik H (2015). MRI of rectal cancer: an overview and update on recent advances. Am J Roentgenol.

[2] Beets-Tan RGH, Lambregts DMJ, Maas M, Bipat S, Barbaro B, Curvo-Semedo L (2018). Magnetic resonance imaging for clinical management of rectal cancer: updated recommendations from the 2016 European Society of Gastrointestinal and Abdominal Radiology (ESGAR) consensus meeting. Eur Radiol.

[3] Moreno CC, Sullivan PS, Mittal PK (2017). MRI evaluation of rectal cancer: staging and restaging. Curr Probl Diagn Radiol.

[4] Kalisz KR, Enzerra MD, Paspulati RM (2019). MRI evaluation of the response of rectal cancer to Neoadjuvant Chemoradiation therapy. RadioGraphics..

[5] Brouwer NPM, Stijns RCH, Lemmens VEPP, Nagtegaal ID, Beets-Tan RGH, Fütterer JJ (2018). Clinical lymph node staging in colorectal cancer; a flip of the coin?. Eur J Surg Oncol.

[6] Van Den Broek JJ, Van Der Wolf FSW, Lahaye MJ, Heijnen LA, Meischl C, Heitbrink MA (2017). Accuracy of MRI in restaging locally advanced rectal cancer after preoperative Chemoradiation. Dis Colon Rectum.

[7] Gröne J, Loch FN, Taupitz M (2018). Schmidt & C, Kreis ME, Schmidt C, et al. accuracy of various lymph node staging criteria in rectal cancer with magnetic resonance imaging. J Gastrointest Surg.

[8] Gao Y, Li J, Ma X, Wang J, Wang B, Tian J (2019). The value of four imaging modalities in diagnosing lymph node involvement in rectal cancer: an overview and adjusted indirect comparison. Clin Exp Med.

[9] Nagtegaal ID, Knijn N, Hugen N, Marshall HC, Sugihara K, Tot T (2017). Tumor deposits in colorectal cancer: improving the value of modern staging-a systematic review and meta-analysis. J Clin Oncol.

[10] Belt EJT, Van Stijn MFM, Bril H, De Lange-De Klerk ESM, Meijer GA, Meijer S (2010). Lymph node negative colorectal cancers with isolated tumor deposits should be classified and treated as stage III. Ann Surg Oncol.

[11] Basnet S, Lou QF, Liu N, Rana R, Shah A, Khadka M (2018). Tumor deposit is an independent prognostic indicator in patients who underwent radical resection for colorectal cancer. J Cancer.

[12] Bouquot M, Creavin B, Goasguen N, Chafai N, Tiret E, André T (2018). Prognostic value and characteristics of N1c colorectal cancer. Color Dis.

[13] Lee DH, Lee JM (2017). Whole-body PET/MRI for colorectal cancer staging: is it the way forward?. J Magn Reson Imaging.

[14] Catalano OA, Masch WR, Catana C, Mahmood U, Sahani DV, Gee MS (2017). An overview of PET/MR, focused on clinical applications. Abdom Radiol.

[15] Catalano OA, Coutinho AM, Sahani DV, Vangel MG, Gee MS, Hahn PF (2017). Colorectal cancer staging: comparison of whole-body PET/CT and PET/MR. Abdom Radiol.

[16] Paspulati RM, Partovi S, Herrmann KA, Krishnamurthi S, Delaney CP, Nguyen NC (2015). Comparison of hybrid FDG PET/MRI compared with PET/CT in colorectal cancer staging and restaging: a pilot study. Abdom Imaging.

[17] Jeong JH, Cho IH, Chun KA, Kong EJ, Kwon SD, Kim JH (2016). Correlation between apparent diffusion coefficients and standardized uptake values in hybrid 18F-FDG PET/MR: preliminary results in rectal cancer. Nucl Med Mol Imaging.

[18] Kang B, Lee JM, Song YS, Woo S, Hur BY, Jeon JH (2016). Added value of integrated whole-body PET/MRI for evaluation of colorectal cancer: comparison with contrast-enhanced MDCT. Am J Roentgenol.

[19] Plodeck V, Rahbari NN, Weitz J, Radosa CG, Laniado M, Hoffmann R-T (2019). FDG-PET/MRI in patients with pelvic recurrence of rectal cancer: first clinical experiences. Eur Radiol.

[20] Bailey JJ, Jordan EJ, Burke C, Ohliger MA, Jane Wang Z, Van Loon K (2018). Does extended PET acquisition in PET/MRI rectal cancer staging improve results?. Am J Roentgenol.

[21] Rutegård MK, Båtsman M, Axelsson J, Brynolfsson P, Brännström F, Rutegård J (2019). PET/MRI and PET/CT hybrid imaging of rectal cancer - description and initial observations from the RECTOPET (REctal cancer trial on PET/MRI/CT) study. Cancer Imaging.

[22] Loughrey M, Quirke P, Shepherd NAN, Hospital GR (2017). Standards and datasets for reporting cancers. R Coll Pathol.

[23] Horne J, Bateman AC, Carr NJ, Ryder I (2014). Lymph node revealing solutions in colorectal cancer: should they be used routinely?. J Clin Pathol.

[24] Bosman FT, Carneiro F, Hruban RH, Theise ND (2010). WHO classification of Tumours of the digestive system.

[25] Sobin L, Gospodarowicz MWC (2009). TNM: classification of malignant tumours.

[26] Regionala cancercentrum i samverkan. Tjock- och ändtarmscancer. NAtionellt vårdprogram. https://www.cancercentrum.se/globalassets/cancerdiagnoser/tjock%2D%2Doch-andtarm-anal/vardprogram/nvpkolorektalcancer_2016-03-15.pdf.

[27] Smith N, Brown G (2008). Preoperative staging of rectal cancer. Acta Oncol.

[28] Kim JH, Beets GL, Kim MJ, Kessels AGHH, Beets-Tan RGHH (2004). High-resolution MR imaging for nodal staging in rectal cancer: are there any criteria in addition to the size?. Eur J Radiol.

[29] Nagtegaal ID, Tot T, Jayne DG, McShane P, Nihlberg A, Marshall HC (2011). Lymph nodes, tumor deposits, and TNM: are we getting better?. J Clin Oncol.

[30] Brown G, Richards CJ, Bourne MW, Newcombe RG, Radcliffe AG, Dallimore NS (2003). Morphologic predictors of lymph node status in rectal cancer with use of high-spatial-resolution MR imaging with histopathologic comparison. Radiology..

[31] Rössler O, Betge J, Harbaum L, Mrak K, Tschmelitsch J, Langner C (2017). Tumor size, tumor location, and antitumor inflammatory response are associated with lymph node size in colorectal cancer patients. Mod Pathol.

[32] Yamaoka Y, Kinugasa Y, Shiomi A, Yamaguchi T, Kagawa H, Yamakawa Y (2017). Preoperative chemoradiotherapy changes the size criterion for predicting lateral lymph node metastasis in lower rectal cancer. Int J Color Dis.

[33] Heijnen LA, Maas M, Beets-Tan RG, Berkhof M, Lambregts DM, Nelemans PJ (2016). Nodal staging in rectal cancer: why is restaging after chemoradiation more accurate than primary nodal staging?. Int J Color Dis.

[34] Stijns R, Philips B, Wauters C, de Wilt J, Nagtegaal I, Scheenen T (2019). Can ex vivo magnetic resonance imaging of rectal cancer specimens improve the Mesorectal lymph node yield for pathological examination?. Investig Radiol.

